# In Search of the Urban Cowboy: The Need to Incorporate Animal Husbandry into the United States Higher Education Curriculum and Its Implications for Production Animal Welfare

**DOI:** 10.3389/fvets.2016.00084

**Published:** 2016-09-21

**Authors:** Courtney Lynd Daigle

**Affiliations:** ^1^Department of Animal Science, Texas A&M University, College Station, TX, USA

**Keywords:** animal husbandry, animal handling, animal welfare, production animal, zoo, higher education, curriculum

The increasingly urban United States population combined with an aging human agricultural population presents a paradigm where there is a need to recruit, retain, and educate younger generations about animal handling and husbandry in production animal agriculture (1). Yet much of the animal husbandry information historically learned through experiences at the family farm must now happen in the classroom. While sustainably meeting the food needs of the future is paramount to a high-quality food system, there is also a need to disseminate generations-old knowledge about how to care for agricultural animals. Agricultural animal welfare science may not make scientific and societal advancements at the same pace as more traditional areas (e.g., nutrition, reproduction) unless we properly train future generations.

There is a need to (1) highlight the importance of incorporating animal husbandry into higher education curriculum and (2) emphasize that professionally training production animal handlers will have positive impact on production animal welfare. Spending more time interacting with agricultural animals will allow students to gain practical animal handling knowledge to enhance animal lives while meeting the food needs of the future. This is the type of curriculum that is needed at the collegiate level – because this is where we will find the urban cowboy.

The human–animal interaction is paramount to good animal welfare, and stockperson attitudes can have a substantial impact on overall animal welfare, animal productivity, and product quality (2–6). New stockpersons may come directly from an undergraduate program with minimal experience directly interacting with livestock. This provides an opportunity to establish best practices and attitudes from the beginning. Irrespective of experience, a positive relationship has been observed between stockperson attitudes and behavior toward animals (7). Teaching stockmanship to students entering the agricultural workforce requires shaping attitude and behavior because a positive attitude toward animals is just as important as receiving a solid foundation of scientific knowledge.

By growing up in cities and suburbs, many students do not have the experiences characterized by growing up on a farm. Enrollment numbers in the United States degree granting animal science programs are steadily growing, are increasingly urban, female, and want to work with animals (8). They are interested in how their food was raised (9) and consider animal welfare an important component of animal production. Teaching animal handling establishes the ideology that low stress handling skills are the norm and animal welfare is paramount to a sustainable food supply. Educators have a responsibility to establish best practices in the classroom that will generate stewardship and sustainability in the industry.

Incorporating animal husbandry into higher education will create transparency to the uninformed university student about the details and common practices important to production agriculture. There is a veil between production agriculture and the general public, and this veil lends itself to mistrust. Much of the information the general public receives about agricultural animals is learned through social media, political movements, and well-funded activists. General knowledge of agricultural animal husbandry may be biased because the public does not understand the reality of daily husbandry practices and the scientific support of their implementation.

This lack of understanding may cause potential animal agriculturists to turn away from production agriculture as an opportunity to engage with animals because they are morally against its implementation. Changing beliefs and attitudes toward animal welfare requires more than a one-off or short-term exposure to these concepts, warranting the need for these concepts to be integrated throughout the entire degree program (10). Teaching husbandry in the classroom provides an opportunity to bridge the gap between the farm and the grocery store. By increasing the public’s understanding of how agricultural animals are managed, we can possibly increase economic gains and the general public’s appreciation of the value of good agricultural animal husbandry.

Animal welfare implies the use of animals and presents a situation where we have entered into a social contract where many humans develop strong bonds with the animals in their care (11). As part of this contract, humans who directly interface with animals have the responsibility to be stewards before businessmen. Husbandry and health choices must be made in the best interest of the animal. Stewards must understand how their actions impact all stages of production because maintenance of the lifelong animal welfare state begins and birth and is sustained through the human–animal interaction. Emphasizing the importance of the stockperson and their role within the animal production system is paramount to ensuring good animal welfare now and in the future (11). Therefore, there is value in teaching animal handling in the classroom as well as increasing our understanding of our actions on animal health, stress, and productivity.

Maintenance of the animal welfare state happens at the animal level. Yet a barrier that urban-born animal science students may perceive to engaging in production agriculture is their lack of experience interacting with agricultural animals. Concepts surrounding biological relevance with regard to housing and husbandry may not be instinctual, which can make understanding and implementation of management practices challenging. From a distance, cattle appear pastoral, but direct contact can be dangerous if animal handlers are not properly trained. Large flocks of hens can be disturbed by quick or irregular movement that could cause smothering and broken bones. Swine can cause injury because they are curious and use their mouths to explore the world around them. Providing targeted opportunities to develop animal handling skills may eliminate perceived barriers to engaging in agriculture.

The curriculum provided in animal science programs does not typically teach basic animal handling and husbandry, as these were skills previous generations were expected to possess upon arrival at the university. A gap exists between what students are learning in the classroom and what is needed in the industry. Students may not have a clear understanding of what is (and is not) needed to provide a good life to food animals. Currently, multiple animal science departments in the United States are undergoing curriculum reviews to address this knowledge gap. Yet identifying strategies to teach this knowledge and meet the requirements of the university are challenging.

But this is an important objective to complete, and students are motivated to engage in experiential learning opportunities (12). The true implementers of good animal welfare are on the ground, interacting with the animals on a daily basis. A positive human–animal interaction and understanding the biology, natural history, and perception of animals are paramount to providing them with a good quality of life (5). Without understanding how our actions are perceived by the animal, we may inadvertently negatively impact the animals in our care. Therefore, the inclusion of animal husbandry and handling to collegiate curriculum is important to the future of animal production and is needed to meet the societal expectations crucial to its social sustainability.

As someone who is from an urban background, has worked in a zoo, and with rural agriculture, opportunities for understanding exist from both sides of the aisle. Annually, over 183 million visitors attend zoos accredited by the Association of Zoos and Aquariums (13). While there is limited opportunity to visit a typical production agricultural farm, the general public can easily access and interact with animals at the zoo – a playground for the urban cowboy.

Zookeeping has become a desirable and competitive occupation (14). Within the last 20 years, the minimum requirements for becoming a zookeeper have risen drastically where now the typical zookeeper holds a Bachelor’s degree or higher (15). The sense of moral duty to engage in an occupation that offers low wages and hard work is reflected in the increased efforts in zoo research, zoo animal training, environmental enrichment, assisted reproductive technology, specialized animal husbandry techniques, and conservation efforts worldwide (16). Yet many of the fundamental requirements of the job and the salaries of zookeepers have not changed (16). What has changed is the public perception of the inherent value of this job.

Society is ready for a similar occupational evolution when it comes to animal handlers in agriculture. Caring for and working with the animals we eat should be an honorable and desirable occupation. If you truly believe in good animal welfare for the animals in our food supply, employment in animal husbandry should be viewed as a privilege to contribute to good animal welfare and to lead by example. Zookeeping may be a “calling,” but the urbanite’s attraction to working with animals can, and should, be extended to agricultural animal care as well.

There is a special kinship between cattlemen and zookeepers. There is an art to both occupations. To be good at either requires patience, confidence, awareness, and a strong gentleness. Being a pen rider is an art. Good riders and good zookeepers are worth their weight in gold for their skill, experience, and finesse. Yet few urbanites know that pen riders exist while many aspire to be zookeepers.

Placing animal agriculture in context can increase understanding and acceptance. For example, feedlots are like boring zoos – the animals are monospecific rather than representative of the earth’s biodiversity. In both scenarios, animal welfare is central to their existence. Both provide high quality feed, water, veterinary treatment, and opportunities to perform most (and in some cases all) of their natural behaviors. They both have a specific purpose. Zoos provide education about biodiversity, conservation, and entertainment. The meat industry provides high quality nutritious protein. Both have value for different reasons but come under similar dynamics of public pressure. Zoos and feedyards are periodically targeted as being cruel, but in reality, they aspire to provide thoughtful and biologically appropriate care.

Differences exist between life as a zoo animal and life as an agricultural animal. Fundamental differences in the economic drivers between the two systems influence management choices. Zoo animals spend most (or all) of their life in the same environment, and zoos typically have a relatively stable animal population. Exhibits are regularly enriched to provide a comfortable environment that is (as close as possible) in alignment with the animal’s biology. Inventories at feedyards are in constant flux. Feedyard cattle are typically juveniles and will have been typically housed on pasture until they have grown large enough to go to the feedyard. Because zoo and feedyard housings have different utility, animal managers place different priorities on environmental management and features. These inherent differences in animal management require understanding their inherently different impacts on animal welfare.

So we return to the search for the urban cowboy. Applied ethologists have been instrumental in implementing change by providing advice to industry and scientific support for relevant welfare concerns (17). While this field is still growing, and the reach of extension agents and researchers trained in animal welfare is expanding, more needs to be done. Expanding the reach and impact of animal welfare science is important, but teaching basic animal handling is fundamental to its implementation. As society works to feed the future with animal welfare as a priority, take the following as food for thought.

Knowing more about the needs and affective states of animals will very likely increase the effectiveness of efforts to improve their welfare. Incorporate animal handling into coursework. Explain what science knows about how animals perceive the world and how this can be different or similar to us. Encourage urban students to intern at feedlots, swine facilities, and hen houses to find their niche. Highlight that providing exceptional care is a pillar of animal agriculture. Engage students in critical and scientific evaluation of the information disseminated on social media. Provide opportunities for students from zoology, ecology, evolution, biology, and wildlife management to become engaged in the food system. Identify accessible analogies that increase understanding of agriculture for students that have not experienced it firsthand.

By providing husbandry knowledge and handling experience in the classroom, we may be able to identify those urban cowboys the future of animal agriculture so desperately needs. Emphasizing opportunities to engage with animals can positively impact animal welfare by teaching appropriate animal handling, animal perception, moral obligations of care, and practical limitations to the next generation of urban-born farmers and ranchers. With this knowledge, future urban cowboys will sustain our food system while providing excellent care to the animals within.

## Author Contributions

CD envisioned, wrote, and edited this manuscript and approved it for publication.

## Conflict of Interest Statement

The author declares that the research was conducted in the absence of any commercial or financial relationships that could be construed as a potential conflict of interest.
